# Hospital Breastfeeding Support during the Early Coronavirus Disease 2019 (COVID-19) Pandemic: Worsening Care for Black, Hispanic, and Asian Mothers

**DOI:** 10.1007/s10995-025-04123-5

**Published:** 2025-07-25

**Authors:** Jane Lazar Tucker, Kimberly Arcoleo, Diane DiTomasso, Brietta M. Oaks, Howard Cabral, Thaís São-João

**Affiliations:** 1 College of Nursing, University of Rhode Island, 350 Eddy St, Providence, RI 02903 USA; 2 College of Health Sciences, University of Rhode Island, 55 Lower College Rd, Kingston, RI 02881 USA; 3 Department of Biostatistics, Boston University School of Public Health, 801 Massachusetts Ave, 3rd floor, Boston, MA 02118 USA

## Abstract

**Objectives:**

This study examines changes in Baby Friendly Hospital Initiative (BFHI) key clinical practices in US hospitals during the early coronavirus disease 2019 (COVID-19) pandemic (April– December 2020) compared to before the pandemic.

**Methods:**

Using data from PRAMS (Pregnancy Risk Assessment Monitoring System) between 2016 and 2020, we conducted linear regression analyses to calculate the percentage-point change in BFHI key clinical practices overall and by race and ethnicity.

**Results:**

A total of 73,380 new mothers were included in our analyses. Overall, receipt of ideal breastfeeding care declined during the pandemic [(pp -1.6, 95% CI (-2.7, -0.4)]. Among racial and ethnic subgroups, declines were noted for Black non-Hispanic [pp -3.4, 95% CI (-6.0, -0.8)], English-speaking Hispanic [pp -3.2, 95% CI (-6.0, -0.4)], Spanish-speaking Hispanic [pp -3.1, 95% CI (-6.1, -0.1)], and Asian/Pacific Islander [pp -4.0, 95% CI (-7.0, -1.0)] mothers; no changes were noted for White non-Hispanic, American Indian/Alaskan Native non-Hispanic, or Mixed Race non-Hispanic respondents.

**Conclusions for Practice:**

The COVID-19 pandemic decreased receipt of the BFHI key clinical practices and exacerbated disparities in evidence-based breastfeeding care for Black non-Hispanic, Hispanic, and Asian/Pacific Islander non-Hispanic mothers. Future research is needed to determine if breastfeeding care has returned to pre-pandemic levels and, if not, to develop strategies to improve breastfeeding care for those most affected by the pandemic.

**Supplementary Information:**

The online version contains supplementary material available at 10.1007/s10995-025-04123-5.

## Significance

What Is Already Known on the Subject?

What this Study Adds?

There is evidence of disparities in receipt of hospital breastfeeding care for Black non-Hispanic, Hispanic, and Asian/Pacific Islander non-Hispanic mothers compared to White non-Hispanic mothers. Preliminary reports suggest that COVID-19 decreased breastfeeding help from hospital staff (Lubbe et al., 2022). No prior study has investigated the possibility of worsening disparities in breastfeeding care during the COVID-19 pandemic.

During the COVID-19 pandemic, Black non-Hispanic, Hispanic, and Asian/Pacific Islander non-Hispanic mothers experienced declining receipt of evidence-based hospital breastfeeding care. No changes in receipt of care were identified for White non-Hispanic, American Indian/Alaskan Native non-Hispanic, or Mixed Race non-Hispanic mothers.

**Supplementary Information:**

The online version contains supplementary material available at 10.1007/s10995-025-04123-5.

## Introduction

High-quality breastfeeding care is associated with significantly higher rates of breastfeeding initiation and duration (Crenshaw & Budin, 2020; Kivlighan et al., 2020; Nelson et al., 2018), but historically, this care has not been received equitably by mothers from different racial and ethnic backgrounds. In a nationally representative analysis of new mothers, it was reported that less than one-quarter of mothers received 100% of the recommended Baby Friendly Hospital Initiative (BFHI) key clinical practices during their delivery hospital stay (Lazar Tucker et al., 2025b). Black non-Hispanic, Hispanic, and Asian/Pacific Islander mothers were less likely to receive ideal breastfeeding care compared to White non-Hispanic mothers. BFHI key clinical practices represent a bundle of evidence-based breastfeeding care that is significantly associated with improved breastfeeding outcomes across all racial and ethnic groups (Barrera et al., 2019; Crenshaw & Budin, 2020; Kivlighan et al., 2020; Nelson et al., 2018; Patterson et al., 2019; Sebastian et al., 2019). Given pre-existing racial and ethnic disparities in breastfeeding care in the US, it is important to identify how the Coronavirus Disease 2019 (COVID-19) pandemic impacted breastfeeding care overall and across racial and ethnic groups because of the possibility of worsening disparities by race and ethnicity.

The COVID-19 pandemic impacted all health service delivery areas, and recent evidence suggests that breastfeeding support was also negatively affected (van Goudoever et al., 2022). Though breastfeeding was eventually determined to be safe in the context of COVID-19, recommendations from health governing bodies in the US were inconsistent with World Health Organization (WHO) guidelines and generally advised against skin-to-skin contact, rooming in, and breastfeeding among mothers who were infected with COVID-19 or had been exposed to the illness (Turner et al., 2022). Few studies have explored the changes to hospital breastfeeding support reported during the pandemic. In one study researchers suggested that COVID-19 resulted in a decrease in breastfeeding help from hospital staff (Lubbe et al., 2022). No published study has investigated the differential impact of the COVID-19 pandemic on breastfeeding support among women from different racial and ethnic backgrounds. Yet in one study of the birthing experiences of Spanish-speaking women during COVID-19, the authors suggested that race and ethnicity could be an important factor; Spanish-speaking women reported that during the pandemic, they lacked an interpreter when they needed one and they were not allowed to have a support person, who might have acted as an interpreter (Granada et al., 2022).

Given the importance of BFHI key clinical practices on breastfeeding outcomes and the mounting evidence of disparities in receipt of this care by race and ethnicity, there is a need to identify the effect of a global health crisis, COVID-19, on the receipt of high-quality breastfeeding care. This study aimed to examine whether the COVID-19 pandemic was associated with changes in receipt of BFHI key clinical practices nationally and whether changes varied by race and ethnicity.

## Methods

### Data

We used 2016–2020 national Pregnancy Risk Assessment Monitoring System (PRAMS) data, an annual population representative survey of new mothers with live births across 45 states. A detailed description of the PRAMS survey methodology is available elsewhere (Shulman et al., 2018). PRAMS participants are sampled from the birth certificate, which allows for robust estimation of nonresponse weights and generalization to the entire population of births within that state (CDC, 2022). This study was designated exempt, and informed consent was waived by the Institutional Review Board at the University of Rhode Island.

### Study Population

We included data from the 27 PRAMS sites that collected information on hospital breastfeeding support. To be eligible for inclusion, mothers had to indicate that they had initiated breastfeeding, a proxy for breastfeeding intention. Only mothers who delivered a full-term (37 weeks or greater) singleton, live birth in a US hospital were included (see Supplemental Fig. 1 for detailed information on inclusion and exclusion criteria).

### Measures

We used self-reported maternal race and ethnicity from the birth certificate and PRAMS survey language (English or Spanish) to create a variable that categorized respondent mothers as non-Hispanic White, non-Hispanic Black, English-speaking Hispanic, Spanish-speaking Hispanic, non-Hispanic Asian/Pacific Islander, non-Hispanic American Indian/Alaskan Native, and non-Hispanic Mixed Race. Hispanic ethnicity was separated into Spanish-speaking and English-speaking because acculturation and nativity are important predictors of breastfeeding outcomes among Hispanic mothers (Bigman et al., 2018; Eilers et al., 2020). BFHI key clinical practices were mapped to the PRAMS survey’s corresponding hospital breastfeeding support questions (see Supplemental Table 1). A summary variable representing the full complement of BFHI key clinical practices received was created as the percent of BFHI key clinical practices a mother reported receiving during her stay, aligned with prior studies (Lazar Tucker et al., 2025a, b). The summary variable was calculated as a percentage to allow for missing data; non-missing data was required for at least five out of eight key clinical practices. The percent of key clinical practices received was broken into the following categories: 0–50%, 51–75%, 76–99%, and 100%. When a mother reported receiving 100% of key clinical practices, she was considered to have received “ideal” breastfeeding care. To investigate the modifying effect of the COVID-19 pandemic on receipt of the BFHI key clinical practices, we created an indicator of whether a respondent mother’s delivery date occurred before the pandemic (2016-February 2020) or during the early pandemic (April-December 2020). We excluded deliveries from March 2020 since it included data from both before COVID-19 and during the pandemic period.

### Statistical Analysis

Data were analyzed using SAS version 9.4 Survey Procedures to account for the complex survey design of PRAMS data. We compared data from the 27 PRAMS sites that collected information on BFHI key clinical practices to the full 51-site PRAMS sample to identify variations in race and ethnicity that would affect generalizability. Descriptive analyses were performed to examine receipt of BFHI key clinical practices by race and ethnicity. Procedures used to examine the data included unweighted frequencies, survey-weighted percents, 95% confidence intervals, and χ^2^ tests. We calculated the percentage point change in receipt of BFHI key clinical practices after the start of the COVID-19 pandemic overall and by race and ethnicity. To do this, we used linear regression models with a binary indicator for receipt of BFHI key clinical practices (1 = received 100% of BFHI key clinical practices, 0 = did not receive) as the outcome and a binary indicator for the pandemic (1 = during the pandemic, 0 = before the pandemic). Results are presented as percentage point changes with 95% confidence intervals and corresponding p values. We set a significance level of 0.05 a priori. Respondent characteristics were compared before COVID-19 and during COVID-19 to identify if any differences would require adjustment for confounding. We followed STROBE (Strengthening the Reporting of Observational Studies in Epidemiology) for reporting observational studies throughout this manuscript (von Elm et al., 2014).

## Results

The 2016–2020 PRAMS survey sample included 202,745 respondent mothers. After applying inclusion and exclusion criteria, the resultant sample was 130,663 respondent mothers, with 73,380 from the 27-site sample that included questions on hospital breastfeeding care (See Supplemental Fig. 1 for details). Respondent mother’s racial and ethnic characteristics were similar in the 27-site sample compared to the whole sample and before versus during the COVID-19 pandemic (See Supplemental Tables 1 and 2).

During the first nine months of the pandemic, respondent mothers reported less receipt of information on breastfeeding from staff [percentage points (pp) -1.0, 95% confidence interval (CI) (-1.7, -0.3)], avoidance of formula supplementation [pp -2.3, 95% CI (-3.8, -0.8)], being advised to breastfeed on demand [pp -1.2, 95% CI (-2.2, -0.1)], and avoiding pacifier use [pp -2.4, 95% CI (-3.9, -0.8)] (See Table 1). Compared to before the pandemic, mothers reported they were more likely to room-in with their infants [pp 1.1, 95% CI (0.4, 1.8)]. Significant declines in individual BFHI key clinical practices were noted among Black non-Hispanic and Asian/Pacific Islander non-Hispanic mothers, with substantial declines in mothers reporting they received information on breastfeeding from staff and help with learning to breastfeed (See Supplemental Table 3). Black non-Hispanic respondents also reported declines in early initiation of breastfeeding and being advised to breastfeed on demand from staff. Asian/Pacific Islander non-Hispanic mothers were less likely to avoid formula supplementation and more likely to room in with their infants.

**Table 1:** Percent of BFHI key clinical practices received before and during coronavirus disease 2019 pandemic (COVID-19), overall

	Before COVID-19	During COVID-19	Difference	
Key Clinical Practices	% (95% CI)	% (95% CI)	pp (95% CI)	*p* value
Step 3: Provided information	95.3 (95.1, 95.6)	94.3 (93.6, 95.0)	-1.0 (-1.7, -0.3)	< 0.01
Step 4: BF within 1st hour	77.5 (77.0, 78.0)	76.8 (75.6, 78.0)	-0.6 (-2.0, 0.7)	0.338
Step 5: Helped learn	84.7 (84.2, 85.1)	83.6 (82.5, 84.6)	-1.1 (-2.3, 0.03)	0.054
Step 6: Only breastmilk	45.7 (45.2, 46.3)	43.4 (42.1, 44.8)	-2.3 (-3.8, -0.8)	< 0.01
Step 7: Rooming in	93.1 (92.8, 93.4)	94.2 (93.5, 94.9)	1.1 (0.4, 1.8)	< 0.01
Step 8: Advised BF on demand	88.5 (88.1, 88.9)	87.3 (86.3, 88.3)	-1.2 (-2.2, -0.1)	< 0.05
Step 9: Did not give pacifier	51.4 (50.8, 52.0)	49.0 (47.6, 50.4)	-2.4 (-3.9, -0.8)	< 0.01
Step 10: Phone # for lactation	79.6 (79.2, 80.1)	79.4 (78.3, 80.6)	-0.2 (-1.5, 1.1)	0.748
Received 100% of steps (ideal care)	19.0 (18.6, 19.5)	17.4 (16.4, 18.5)	-1.6 (-2.7, -0.4)	< 0.01

Abbreviation: BFHI, Baby Friendly Hospital Initiative; pp, percentage point

**Fig. 1:**
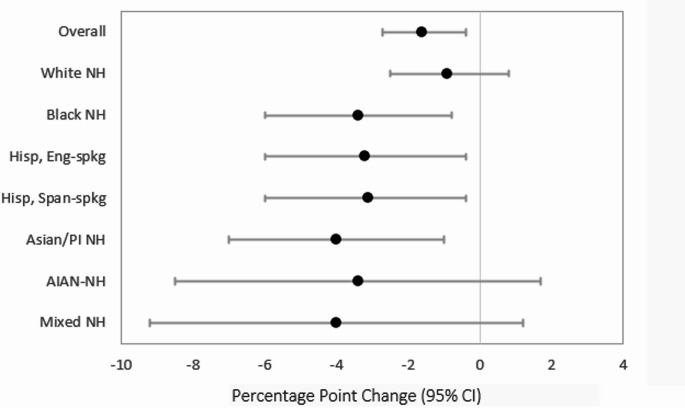
Change in percent of mothers receiving 100% of BFHI key clinical practices after the start of the coronavirus disease 2019 (COVID-19) pandemic in the overall sample of new mothers, and by racial and ethnic subgroup, PRAMS (Pregnancy Risk Assessment Monitoring System) data, January 2016-December 2020. Error bars represent 95% CIs

Figure 1 shows the change in the percentage of respondents reporting they received 100% of BFHI key clinical practices (ideal breastfeeding care). Overall, receipt of ideal breastfeeding care declined during the pandemic [(pp -1.6, 95% CI (-2.7, -0.4)]. Significant declines were noted for Black non-Hispanic [pp -3.4, 95% CI (-6.0, -0.8)], English-speaking Hispanic [pp -3.2, 95% CI (-6.0, -0.4)], Spanish-speaking Hispanic [pp -3.1, 95% CI (-6.1, -0.1)], and Asian/Pacific Islander [pp -4.0, 95% CI (-7.0, -1.0)] mothers; no changes were noted for White non-Hispanic, American Indian/Alaskan Native non-Hispanic, or Mixed Race non-Hispanic respondents.

## Conclusions for Practice

This study provides a nearly nationally representative picture of changes in evidence-based breastfeeding care before and during the early COVID-19 pandemic. Overall, these results indicate that the COVID-19 pandemic decreased receipt of the BFHI key clinical practices and exacerbated disparities by race and ethnicity, with Black non-Hispanic, Hispanic, and Asian/Pacific Islander non-Hispanic mothers experiencing statistically significant declines in receipt of ideal breastfeeding care, while White non-Hispanic mothers did not. This finding is important because prior research has shown that among mothers who receive 100% of key clinical practices, racial and ethic disparities in continued breastfeeding to 10 weeks is eliminated (Lazar Tucker et al., 2025). Therefore, it is likely that decreased receipt of high-quality breastfeeding support during the early COVID-19 pandemic also resulted in increased racial and ethnic disparities in breastfeeding continuation.

In the early days of the COVID-19 pandemic, hospital staff were uncertain and fearful about the novel coronavirus. Healthcare workers were not provided adequate personal protective equipment (Cohen & Rodgers, 2020) and were often afraid of contracting COVID-19 and spreading the illness to their family members (Coelho et al., 2020). Breastfeeding care requires nursing staff to be in close contact with new mothers to assist them with positioning and latching the baby. One possible explanation for the decline in breastfeeding support during the early pandemic is that nursing staff feared getting close to patients because of their fear of contracting COVID-19.

Black non-Hispanic and Hispanic people experienced higher rates of COVID-19 than White non-Hispanic women, due to an increased exposure risk because they are more likely to work in jobs that cannot be done remotely, to live in larger households, and to take public transportation to work (Hill & Artiga, 2022). A higher risk of COVID-19 exposure and illness could partially explain the differences in receipt of hospital breastfeeding care among Black and Hispanic mothers. As mentioned, recommendations regarding breastfeeding and breastfeeding care were unclear early in the pandemic, resulting in hospital policies that discouraged breastfeeding among mothers who had COVID-19 or had been exposed to COVID-19.

It is also possible that Black and Hispanic women could have been stigmatized as more likely to be at risk of COVID-19 resulting in an avoidance of close contact care because nursing staff feared contracting COVID-19. This stigma was likely compounded by preexisting race-based discrimination resulting in historically unequal breastfeeding care provided to patients of color (Thomas, 2018).

Asian people were not noted to have increased risk of COVID-19 exposure or illness (Hill & Artiga, 2022) but they were the target of racism and hate crimes early in the pandemic as a result of politicians and social media platforms spreading anti-Asian rhetoric (Human Rights Watch, 2020). It is possible that the decline in breastfeeding care among Asian mothers was due to stigma and discrimination by healthcare staff. While there are no published studies to date that have documented healthcare stigma against Asian people during the COVID-19-19 pandemic, race-related violence and hate crimes against Asian Americans and Asian-looking people increased by 339% during the pandemic (Levin, 2021). In response to the concerning increase in anti-Asian rhetoric and violence in 2020, the American Medical Association declared racism against Asian Americans a public health crisis (O’Reilly, 2021). Though we cannot say with certainty the cause of the decline in breastfeeding care for Asian/Pacific Islander non-Hispanic mothers in the early pandemic, this finding is concerning and warrants further study.

This study has several limitations. It relied on self-reported data, which could be subject to response bias. This study was limited by the short duration of pandemic data included, without information on how breastfeeding care changed further into the pandemic, or post-pandemic. As mentioned, we can only describe mothers’ reported experience with the breastfeeding care they received; we cannot ask women or their healthcare providers their perceptions of why they experienced fewer key clinical practices that support breastfeeding during the pandemic. However, future research could explore these topics. Finally, while the respondent characteristics were similar before the COVID-19 pandemic and during COVID-19, there could be residual confounding that was not accounted for in these analyses. Future research studies could explore impact of the respondent mothers’ sociodemographic characteristics within racial and ethnic group.

Despite its limitations, this study makes important contributions to our understanding of changes in evidence-based hospital breastfeeding care during the early COVID-19 pandemic and worsening disparities by race and ethnicity. These findings point toward a need for future studies that investigate whether levels of BFHI key clinical practices have returned to pre-pandemic levels, or if more work needs to be done to re-establish pre-pandemic evidence-based breastfeeding care in US hospitals for Black non-Hispanic, Hispanic and Asian non-Hispanic women. In addition, as the threat of a future pandemic is ever-present, identification of strategies to make breastfeeding care more resistant to the next pandemic is warranted.

## Electronic Supplementary Material

Below is the link to the electronic supplementary material.

Supplementary Material 1

## Data Availability

The data are available from the Pregnancy Risk Assessment Monitoring System (PRAMS) working group and the Centers for Disease Control and Prevention.

## Code Availability

Data cleaning and analysis code is available upon request from the corresponding author (JLT).

## Declarations

### Study Registration

osf.io/3rb7c [OSF link]. This statistical analysis plan is available online: osf.io/3rb7c [OSF link]
