# Supplementary material for: Hospital Breastfeeding Support during the Early Coronavirus Disease 2019 (COVID-19) Pandemic: Worsening Care for Black, Hispanic, and Asian Mothers
Source: Matern Child Health J. 2025 Jul 25;29(9):1226–31. doi: 10.1007/s10995-025-04123-5 (PMC12460545; doi:10.1007/s10995-025-04123-5)

Hospital Breastfeeding Support During the Coronavirus Disease 2019 (COVID-19) Pandemic: Worsening Care for Black, Hispanic, and Asian Mothers

# Supplemental Table 1: Characteristics of respondents from 27-site and full samples

| Characteristics | 27-site sample | | | Full sample | | |
| --- | --- | --- | --- | --- | --- | --- |
|  | N | % | 95% CI | N | % | 95% CI |
| Total | 73,380 | 100 |  | 130,663 | 100 |  |
| Maternal race and ethnicity | | | | | | |
| White NH | 33,774 | 56.6 | (56.1, 57.1) | 61,425 | 58.4 | (58.0, 58.8) |
| Black NH | 10,251 | 13.2 | (12,8, 13.5) | 21,149 | 13.2 | (13.0, 13.5) |
| Hispanic-Eng | 9,716 | 11.6 | (11.2, 11.9) | 15,230 | 10.9 | (10.7, 11.2) |
| Hispanic-Span | 5,673 | 9.2 | (8.9, 9.5) | 10,083 | 8.4 | (8.1, 8.6) |
| Asian/PI NH | 6,076 | 6.0 | (5.8, 6.3) | 10,500 | 5.9 | (5.8, 6.1) |
| AI/AN NH | 3,803 | 1.0 | (1.0, 1.1) | 5,482 | 0.8 | (0.7, 0.8) |
| Mixed Race NH | 4,087 | 2.4 | (2.2, 2.5) | 6,794 | 2.4 | (2.3, 2.5) |

*27 sites included questions about hospital practices that support breastfeeding; Ns are unweighted, percentages are weighted

# Supplemental Table 2: Characteristics of respondents from 27-site and full samples before and during Covid-19

|  | 27-site sample | | | | | | Full Sample | | | | | |  |
| --- | --- | --- | --- | --- | --- | --- | --- | --- | --- | --- | --- | --- | --- |
|  | Before Covid-19 | | | During Covid-19 | | | Before Covid-19 | | | During Covid-19 | | |  |
| Characteristics | N | % | 95% CI | N | % | 95% CI | N | % | 95% CI | N | % | 95% CI |  |
| Total | 62,438 | 100 | 10,942 | 10,942 | 100 |  | 109,953 | 100 |  | 20,710 | 100 |  |  |
| Maternal race and ethnicity | | | | | | | | | | | | | |
| White NH | 28,971 | 56.2 | (55.7, 56.7) | 4,803 | 59.2 | (58.0, 60.4) | 52,088 | 58.4 | (58.0, 58.8) | 9,337 | 58.2 | (57.3, 59.1) |  |
| Black NH | 8,842 | 13.3 | (12.9, 13.7) | 1,409 | 12.5 | (11.7, 13.4) | 17,917 | 13.3 | (13.0, 13.6) | 3,232 | 12.6 | (12.0, 13.2) |  |
| Hisp-English | 8,139 | 11.6 | (11.2, 12.0) | 1,577 | 11.3 | (10.5, 12.1) | 12,623 | 10.7 | (10.4, 11.0) | 2,607 | 12.1 | (11.4, 12.8) |  |
| Hisp-Spanish | 4,926 | 9.5 | (9.1, 9.8) | 747 | 7.5 | (6.8, 8.3) | 8,708 | 8.5 | (8.2, 8.7) | 1,375 | 7.9 | (7.2, 8.5) |  |
| Asian/PI NH | 5,041 | 6.1 | (5.8, 6.3) | 1,035 | 5.9 | (5.4, 6.5) | 8,634 | 6.0 | (5.8, 6.1) | 1,866 | 5.8 | (5.4, 6.2) |  |
| AI/AN NH | 3,152 | 1.1 | (1.0, 1.1) | 651 | 0.9 | (0.8, 1.1) | 4,488 | 0.8 | (0.7, 0.8) | 994 | 0.8 | (0.7, 1.0) |  |
| Mixed Race NH | 3,367 | 2.3 | (2.2, 2.5) | 720 | 2.6 | (2.2, 3.0) | 5,495 | 2.4 | (2.2, 2.5) | 1,299 | 2.6 | (2.3, 2.8) |  |

Notes: 27 sites included questions about hospital practices that support breastfeeding, Ns are unweighted, percentages are weighted, Abbreviations: NH = non-Hispanic, AI = American Indian, AN = Alaska Native

# Supplemental Table 3: BFHI key clinical practices by race and ethnicity before and during Covid-19

|  | | Before Covid-19 | During Covid-19 | Difference | | |
| --- | --- | --- | --- | --- | --- | --- |
|  | | % (95% CI) | % (95% CI) | pp (95% CI) | *p* value |  |
| Step 3: Provided information | | | | | | |
|  | Overall | 95.3 (95.1, 95.6) | 94.3 (93.6, 95.0) | -1.0 (-1.7, -0.3) | <0.01 |  |
|  | White NH | 96.0 (95.7, 96.3) | 95.3 (94.4, 96.1) | -0.7 (-1.6, 0.2) | 0.119 |  |
|  | Black NH | 95.4 (94.6, 96.1) | 92.2 (89.9, 94.4) | -3.2 (-5.6, -0.8) | <0.01 |  |
|  | Hispanic-Eng | 95.0 (94.2, 96.0) | 92.5 (90.3, 94.8) | -2.4 (-4.8, -0.1) | <0.05 |  |
|  | Hispanic-Span | 91.1 (89.8, 92.3) | 92.7 (89.3, 96.1) | 1.6 (-2.0, 5.2) | 0.417 |  |
|  | Asian/PI NH | 96.5 (95.8, 97.2) | 94.2 (91.9, 96.5) | -2.3 (-4.7, 0.1) | <0.05 |  |
|  | AI/AN NH | 93.5 (91.6, 95.5) | 93.6 (90.1, 97.0) | 0.03 (-4.0, 4.1) | 0.990 |  |
|  | Mixed Race NH | 95.8 (94.5, 97.2) | 96.2 (94.0, 98.5) | 0.4 (-2.2, 2.9) | 0.775 |  |
| Step 4: BF w/in 1^st^ hour | | | | | | |
|  | Overall | 77.5 (77.0, 78.0) | 76.8 (75.6, 78.0) | -0.6 (-2.0, 0.7) | 0.338 |  |
|  | White NH | 80.7 (80.1, 81.3) | 80.1 (78.5, 81.6) | -0.6 (-2.3, 1.0) | 0.461 |  |
|  | Black NH | 71.1 (69.5, 72.7) | 66.2 (62.2, 70.2) | -4.9 (-9.2, -0.6) | <0.05 |  |
|  | Hispanic-Eng | 75.3 (73.7, 76.9) | 76.5 (73.2, 79.7) | 1.2 (-2.5, 4.9) | 0.525 |  |
|  | Hispanic-Span | 74.3 (72.3, 76.2) | 76.9 (72.2, 81.6) | 2.6 (-2.5, 7.8) | 0.322 |  |
|  | Asian/PI NH | 70.0 (67.9, 72.1) | 66.9 (61.8, 72.0) | -3.1 (-8.7, 2.5) | 0.266 |  |
|  | AI/AN NH | 81.6 (78.7, 84.5) | 85.4 (81.5, 89.3) | 3.8 (-0.9, 8.5) | 0.136 |  |
|  | Mixed Race NH | 77.8 (74.8, 80.8) | 75.3 (67.7, 82.8) | -2.6 (-10.9, 5.7) | 0.528 |  |
| Step 5: Helped learn | | | | | | |
|  | Overall | 84.7 (84.2, 85.1) | 83.6 (82.5, 84.6) | -1.1 (-2.3, 0.03) | 0.054 |  |
|  | White NH | 83.6 (83.1, 84.2) | 83.4 (82.0, 84.8) | -0.2 (-1.7, 1.3) | 0.767 |  |
|  | Black NH | 86.0 (84.7, 87.2) | 80.1 (76.7, 83.5) | -5.9 (-9.4, -2.3) | <0.001 |  |
|  | Hispanic-Eng | 86.2 (85.0, 87.5) | 85.8 (83.2, 88.3) | -0.5 (-3.3, 2.4) | 0.746 |  |
|  | Hispanic-Span | 83.3 (81.8, 84.9) | 84.8 (80.6, 89.1) | 1.5 (-3.1, 6.0) | 0.541 |  |
|  | Asian/PI NH | 91.9 (90.7, 93.1) | 87.5 (83.7, 91.3) | -4.4 (-8.2, -0.6) | <0.05 |  |
|  | AI/AN NH | 79.2 (76.6, 81.7) | 79.4 (72.7, 86.0) | 0.2 (-7.6, 8.0) | 0.958 |  |
|  | Mixed Race NH | 83.4 (80.8, 85.9) | 82.7 (76.4, 89.0) | -0.7 (-7.8, 6.5) | 0.846 |  |
| Step 6: Only breastmilk | | | | | | |
|  | Overall | 45.7 (45.2, 46.3) | 43.4 (42.1, 44.8) | -2.3 (-3.8, -0.8) | <0.01 |  |
|  | White NH | 54.5 (53.8, 55.3) | 52.2 (50.4, 54.0) | -2.3 (-4.3, -0.4) | <0.05 |  |
|  | Black NH | 31.0 (29.4, 32.7) | 27.3 (23.7, 31.0) | -3.7 (-7.7, 0.3) | 0.080 |  |
|  | Hispanic-Eng | 40.7 (38.9, 42.5) | 37.9 (43.2, 41.5) | -2.9 (-6.9, 1.2) | 0.175 |  |
|  | Hispanic-Span | 28.4 (26.5, 30.3) | 24.1 (19.6, 28.6) | -4.3 (-9.2, 0.6) | 0.099 |  |
|  | Asian/PI NH | 31.7 (29.6, 33.9) | 25.0 (21.0, 29.1) | -6.7 (-11.2, -2.1) | <0.01 |  |
|  | AI/AN NH | 47.1 (44.0, 50.2) | 42.9 (35.2, 50.6) | -4.2 (-12.5, 4.1) | 0.327 |  |
|  | Mixed Race NH | 48.2 (44.8, 51.5) | 43.9 (35.9, 51.9) | -4.3 (-11.4, 2.8) | 0.342 |  |
| Step 7: Rooming in | | | | | | |
|  | Overall | 93.1 (92.8, 93.4) | 94.2 (93.5, 94.9) | 1.1 (0.4, 1.8) | <0.01 |  |
|  | White NH | 93.2 (92.8, 93.6) | 94.3 (93.4, 95.2) | 1.0 (0.1, 2.0) | 0.053 |  |
|  | Black NH | 93.5 (92.7, 94.3) | 93.0 (90.9, 95.0) | -0.5 (-2.8, 1.7) | 0.617 |  |
|  | Hispanic-Eng | 93.8 (93.0, 94.7) | 95.0 (93.3, 96.7) | 1.2 (-0.7, 3.1) | 0.272 |  |
|  | Hispanic-Span | 92.4 (91.3, 93.5) | 93.2 (90.5, 95.9) | 0.8 (-2.2, 3.7) | 0.612 |  |
|  | Asian/PI NH | 90.1 (88.6, 91.5) | 94.6 (92.5, 96.6) | 4.5 (2.0, 7.0) | <0.01 |  |
|  | AI/AN NH | 96.3 (95.4, 97.2) | 97.7 (96.4 99.1) | 1.4 (-0.2, 3.1) | 0.137 |  |
|  | Mixed Race NH | 93.2 (91.4, 95.1) | 96.3 (94.0, 98.5) | 3.0 (0.04, 6.0) | 0.071 |  |
| Step 8: Staff advised BF on demand | | | | | | |
|  | Overall | 88.5 (88.1, 88.9) | 87.3 (86.3, 88.3) | -1.2 (-2.2, -0.1) | <0.05 |  |
|  | White NH | 90.0 (89.5, 90.4) | 89.0 (87.8, 90.2) | -1.0 (-2.3, 0.3) | 0.126 |  |
|  | Black NH | 86.4 (85.1, 87.6) | 82.4 (79.1, 85.8) | -3.9 (-7.4, -0.4) | <0.05 |  |
|  | Hispanic-Eng | 85.6 (84.2, 87.0) | 85.6 (83.0, 88.2) | -0.0 (-3.0, 2.9) | 0.988 |  |
|  | Hispanic-Span | 86.7 (85.2, 88.2) | 85.4 (81.1, 89.6) | -1.3 (-5.9, 3.2) | 0.552 |  |
|  | Asian/PI NH | 89.0 (87.7, 90.3) | 85.5 (81.4, 89.6) | -3.5 (-7.8, 0.8) | 0.081 |  |
|  | AI/AN NH | 86.9 (84.2, 89.6) | 90.6 (87.4, 93.8) | 3.6 (-0.4, 7.7) | 0.098 |  |
|  | Mixed Race NH | 86.4 (83.6, 89.1) | 89.1 (84.2, 94.0) | 2.7 (-2.8, 8.2) | 0.370 |  |
| Step 9: Did not give pacifier | | | | | | |
|  | Overall | 51.4 (50.8, 52.0) | 49.0 (47.6, 50.4) | -2.4 (-3.9, -0.8) | <0.01 |  |
|  | White NH | 52.9 (52.1, 53.7) | 51.7 (49.8, 53.6) | -1.2 (-3.3, 0.8) | 0.253 |  |
|  | Black NH | 46.7 (44.8, 48.5) | 42.9 (38.7, 47.0) | -3.8 (-8.3, 0.7) | 0.103 |  |
|  | Hispanic-Eng | 50.5 (48.6, 52.4) | 45.4 (41.5, 49.3) | -5.1 (-9.4, -0.7) | <0.05 |  |
|  | Hispanic-Span | 50.6 (48.4, 52.9) | 47.1 (41.4, 52.7) | -3.5 (-9.5, 2.4) | 0.255 |  |
|  | Asian/PI NH | 51.8 (49.5, 54.2) | 50.2 (44.8, 55.6) | -1.6 (-7.5, 4.2) | 0.584 |  |
|  | AI/AN NH | 45.9 (41.9, 49.9) | 46.0 (38.1, 53.8) | 0.0 (-7.1, 7.3) | 0.984 |  |
|  | Mixed Race NH | 49.7 (45.9, 53.5) | 36.2 (28.7, 43.7) | -13.5 (-21.0, -6.1) | <0.01 |  |
| Step 10: Given phone # for lactation | | | | | | |
|  | Overall | 79.6 (79.2, 80.1) | 79.4 (78.3, 80.6) | -0.2 (-1.5, 1.1) | 0.748 |  |
|  | White NH | 82.0 (81.4, 82.6) | 82.0 (80.5, 83.5) | 0.0 (-1.6, 1.6) | 0.972 |  |
|  | Black NH | 80.4 (79.0, 81.8) | 80.7 (77.6, 83.8) | 0.3 (-3.1, 3.7) | 0.855 |  |
|  | Hispanic-Eng | 78.5 (77.0, 80.1) | 76.2 (72.8, 79.6) | -2.3 (-6.1, 1.5) | 0.220 |  |
|  | Hispanic-Span | 65.7 (63.6, 67.7) | 61.0 (55.4, 66.6) | -4.7 (-10.7, 1.3) | 0.122 |  |
|  | Asian/PI NH | 80.0 (78.3, 81.7) | 78.2 (73.4, 82.9) | -1.9 (-7.0, 3.3) | 0.465 |  |
|  | AI/AN NH | 76.5 (73.8, 79.1) | 69.9 (61.8, 77.9) | -6.6 (-15.8, 2.6) | 0.105 |  |
|  | Mixed Race NH | 80.9 (78.1, 83.7) | 87.6 (83.9, 91.3) | 6.7 (2.4, 11.0) | <0.01 |  |
| Received 100% of steps: “ideal BF care” | | | | | | |
|  | Overall | 19.0 (18.6, 19.5) | 17.4 (16.4, 18.5) | -1.6 (-2.7, -0.4) | <0.01 |  |
|  | White NH | 22.9 (22.3, 23.5) | 22.0 (20.5, 23.6) | -0.9 (-2.5, 0.8) | 0.319 |  |
|  | Black NH | 12.6 (11.4, 13.8) | 9.2 (6.8, 11.5) | -3.4 (-6.0, -0.8) | <0.05 |  |
|  | Hispanic-Eng | 16.4 (15.1, 17.7) | 13.2 (10.8, 15.7) | -3.2 (-6.0, -0.4) | <0.05 |  |
|  | Hispanic-Span | 11.1 (9.7, 12.5) | 8.0 (5.3, 10.6) | -3.1 (-6.1, -0.1) | <0.05 |  |
|  | Asian/PI NH | 13.9 (12.2, 15.6) | 9.9 (7.4, 12.4) | -4.0 (-7.0, -1.0) | <0.05 |  |
|  | AI/AN NH | 18.6 (16.3, 20.9) | 15.2 (10.6, 19.8) | -3.4 (-8.5, 1.7) | 0.225 |  |
|  | Mixed Race NH | 20.4 (18.0, 23.0) | 16.4 (11.4, 21.4) | -4.0 (-9.2, 1.2) | 0.154 |  |

*Percentages are weighted, Abbreviations: NH =non-Hispanic, AI = American Indian, AN = Alaska Native, pp = percentage point difference, BF = breastfeeding

# Supplemental Figure 1: Flow chart of study population selection including inclusion/exclusion criteria


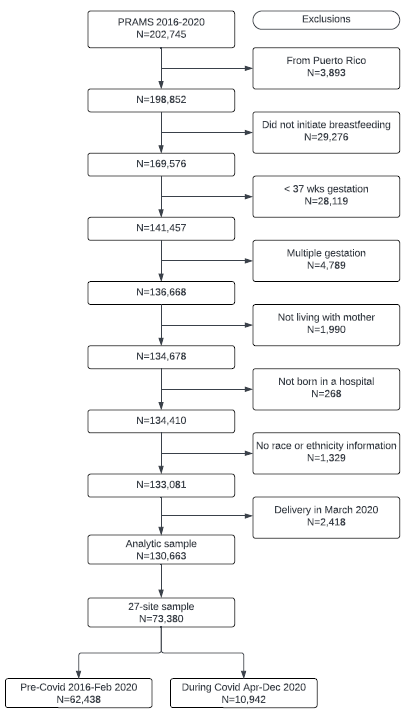

Supplement: Supplementary file 1 — Supplementary Material 1 [file 10995_2025_4123_MOESM1_ESM.docx]
